# Silk fibroin/hydroxyapatite scaffold: a highly compatible material for bone regeneration

**DOI:** 10.1080/14686996.2020.1748520

**Published:** 2020-04-30

**Authors:** Muhammad Saleem, Sidra Rasheed, Chen Yougen

**Affiliations:** a Institute for Advanced Study, Shenzhen University, Nanshan District, Shenzhen, Guangdong, 518060, China; b Department of Optoelectronic Science and Technology, 518060, Shenzhen University, P.R China; c Department of Chemistry, University of Kotli, Azad Jammu and Kashmir; d Interdisciplinary Research Centre in Biomedical Materials, COMSATS Institute of Information Technology, Defence Road, Off. Raiwind Road, Lahore, 54000, Pakistan

**Keywords:** Silk fibroin, hydroxyapatite, scaffold, bone regeneration, biomaterials, 103 Composites, 102 Porous / Nanoporous / Nanostructured materials, 211 Scaffold / Tissue engineering/Drug delivery, 301 Chemical syntheses / processing

## Abstract

In recent years remarkable efforts have been made to produce artificial bone through tissue engineering techniques. Silk fibroin (SF) and hydroxyapatite (HA) have been used in bone tissue regeneration as biomaterials due to mechanical properties of SF and biocompatibility of HA. There has been growing interest in developing SF/HA composites to reduce bone defects. In this regard, several attempts have been made to study the biocompatibility and osteoconductive properties of this material. This article overviews the recent advance from last few decades in terms of the preparative methods and application of SF/HA in bone regeneration. Its first part is related to SF that presents the most common sources, preparation methods and comparison of SF with other biomaterials. The second part illustrates the importance of HA by providing information about its production and properties. The third part presents comparative studies of SF/HA composites with different concentrations of HA along with methods of preparation of composites and their applications.

## Graphical abstract

**Figure:**
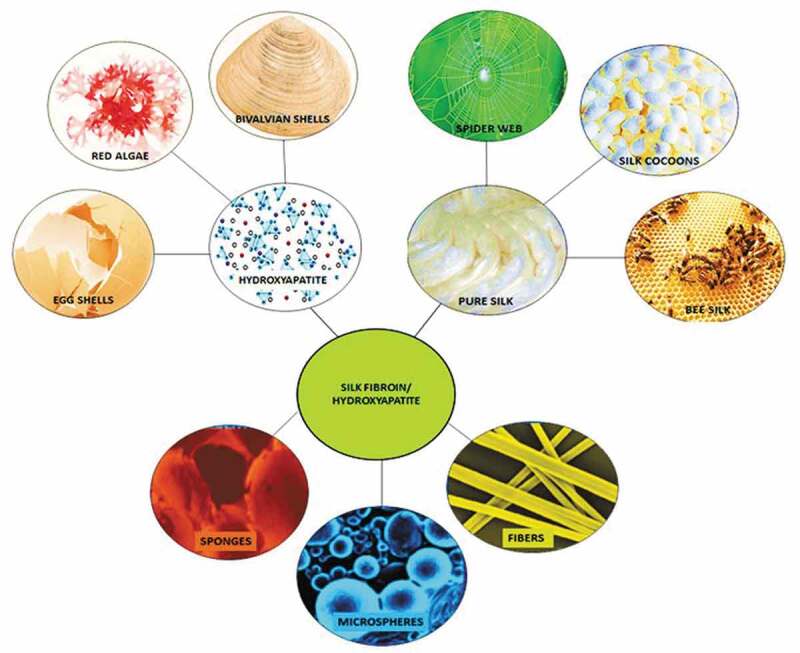

## 1. Introduction

Bone is one of the most important and active connective tissues in human body. It is continuously redesigned to bear loads and to quickly heal injuries. Bone is mainly composed of cells, fibres, collagen and hydroxyapatite [1]. Bone fractures and large bone defects occur due to different types of injuries. Healing rate is directly related to bone defect size. Fibrous tissues migrate very rapidly in a repairing process if bone defect is of large size. Materials which are considered to be the ‘gold standards’ in bone repairing are autografts and allografts [2]. When a tissue or organ is transplanted from one part to another within the same body, it is called autograft, while the transplant of an organ or tissue from one individual to another of the same species is known as allograft. However, both procedures have their own pros and cons. For instance, limitation in donor site and secondary operation requirements are drawbacks in autograft, while health issues like HIV and hepatitis are the associated problems in allografts. Therefore, some replaceable biocompatible materials available *in vivo* are greatly desired to overcome the bone defects. In this aspect, tissue engineering is playing an important role. Various biomaterials such as bioglass (Na_2_O–CaO–SiO_2_–P_2_O_5_), sintered tricalcium phosphate (Ca_3_(PO_4_)_2_), wollastonite (CaO_·_SiO_2_), MgO–CaO–SiO_2_ glassy matrix and hydroxyapatite (Ca_10_(PO_4_)_6_(OH)_2_, HA) are being studied for their applications in bone regeneration. A good bone implant material should have porous structure, mechanical strength, and close resemblance to the framework of extracellular matrix of bone [3,4]. It should also have temporary architecture for attachment and proliferation of bone forming cells for bone regeneration [5,6]. Among the above-mentioned materials, a silk fibroin/hydroxyapatite (SF/HA) composite shows all these characteristics that favours its use in bone implants.

**Figure 1.**
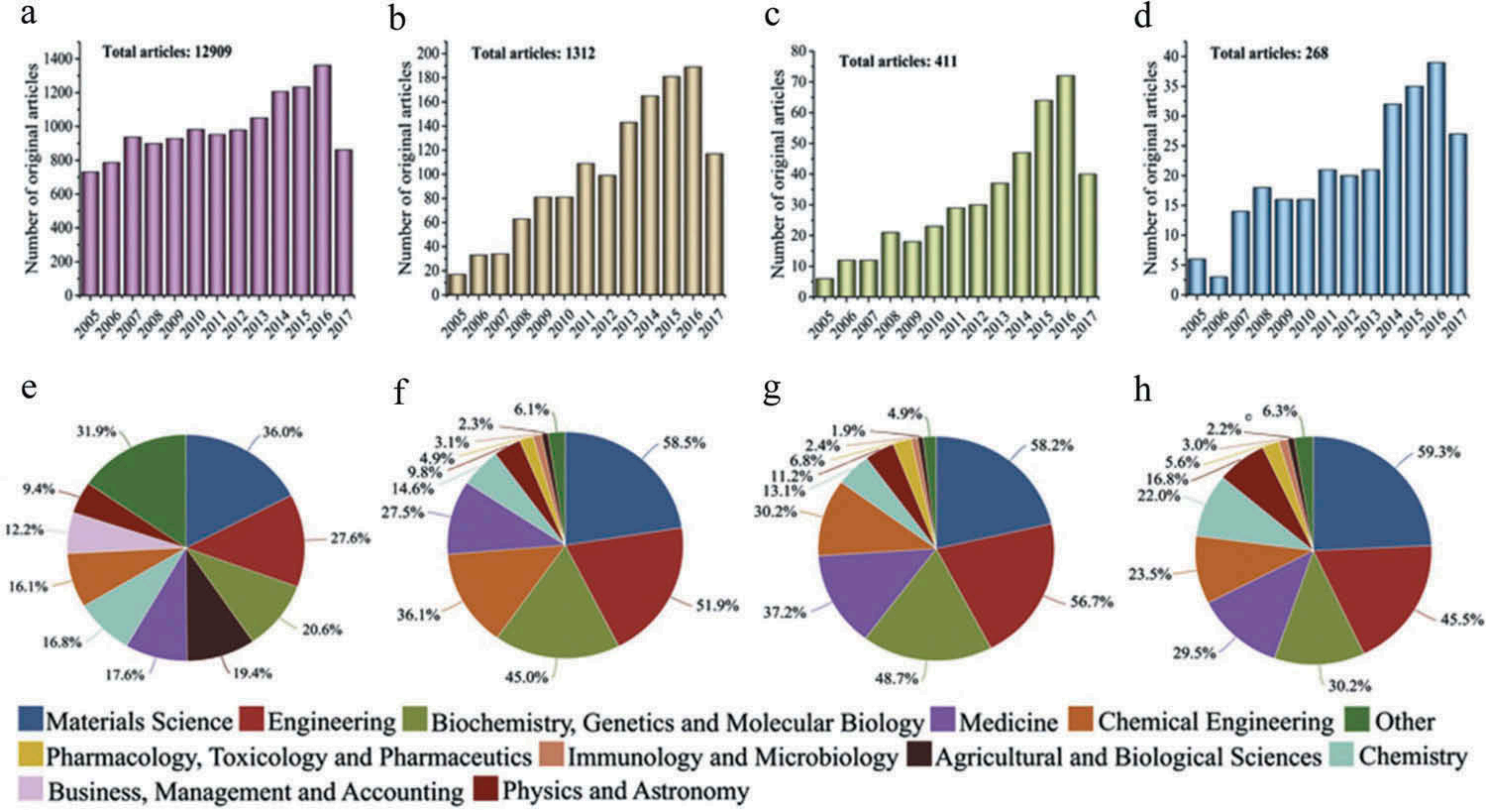
Publication frequency of SF and applications of SF as a biomaterial based on Scopus database: (a) SF-related publications, (b) SF used for tissue engineering, (c) SF used for bone tissue engineering, (d) SF/HA composites, and (e) to (h) remarkable studies related to parts (a) to (d) [7]

On one hand, SF is gaining considerable attention in the field of tissue engineering due to its high mechanical strength and biocompatibility. Its β-sheet structure makes itself easily processable and convertible into various structures, such as hydrogels, fibres, membranes and microsphere. Utilization of SF in blood vessels, bone, cartilage and skin has been studied by tissue engineering for years, and such use has been expending tremendously, as witnessed from the trend of publications during last few decades (Figure 1) [7,8]. SF aids cell activity without causing any destruction to immune system by substituting nutrients and cell growth factors due to the porosity caused by its β-sheet structure. The above-mentioned properties make SF an utmost reliable material in bone regeneration. Moreover, bone implant material should have osteoconductivity, so that the cells responsible for new bone regeneration can adhere to it.

On the other hand, HA is the major inorganic component of human bone tissue. It exhibits high biocompatibility, bioactivity, and bone formability, and thus shows chemical interaction with host cells. It has been considered to be one of the most potential materials for bone implant due to its osteoconductive and osteointegrative ability. However, there are still some downsides of HA, such as insufficient mechanical strength and brittleness that limit its application in clinical use [9]. In last few decades, many efforts have been made to prepare HA/polymer composites to improve such drawbacks. For instance, collagen, chitosan, and gelatine are typically employed bio-based polymers. The limitations of HA can be overcome by incorporating with various biomaterials. In particular, when SF is incorporated with HA, a promising composite biomaterial with tougher mechanical property is produced for bone engineering.

An effective regenerative material for bone tissue needs three dimensional (3D) porous structures with osteogenic properties [10]. As a result, silk and its different forms are suitable candidates and have attracted growing attention as a matrix materials owing to its outstanding biocompatibility, slow degradation rate, and tremendous mechanical strength. However, to increase its osteoconductivity and inductivity, the accumulation of osteoinductive features is necessary [11]. Precisely for this purpose, the use of inorganic/organic composites has been extensively explored. HA or bioglass micro/nanoparticles have been embedded within the scaffold walls to achieve the osteoconductivity of SF scaffolds. However, it remains challenges to optimize the silk-based scaffolds with satisfied consequence. Perhaps one of the most challenging issues is the uniform dispersion of inorganic powders within polymer matrix, due to the inherent aggregation propensity of the powders [12,13]. Several attempts have been made to disperse calcium phosphate (CaP) particles inside SF scaffolds. For example, Kim et al. develop salt‐leached SF scaffolds, followed by the formation of CaP crystals on the scaffold surface [14]. Zhang et al. prepare SF/CaP hybrid powder with enhanced distribution homogeneity to attain improved osteogenic differentiation of bone mesenchymal stem cells (BMSCs) [13]. Nevertheless, these types of hybrids can be uniformly distributed within the SF scaffolds at macroscopic scale, but they get aggregated at microscopic level. In another interesting effort, Yan et al. used combination approach, which involves an in situ synthesis technique and the use of highly concentrated SF solution for the formation of nano‐sized CaP particles to prevent the aggregation of the particles [15]. Even though they claimed the homogeneous distribution of CaP nanoparticles at both macroscopic and microscopic level, highly concentrated SF solution cannot completely prohibit the self-assembly of CaP nanoparticles. In order to attain homogeneous nanoparticle distribution at nanometre scales, SF/HA scaffolds with better osteoconductive property are therefore used [16]. This review mainly focuses on the preparation, crystal structure, and properties of SF/HA scaffold as one of the highly compatible materials for bone regeneration.

## 2. Silk fibroin

**Figure 2.**
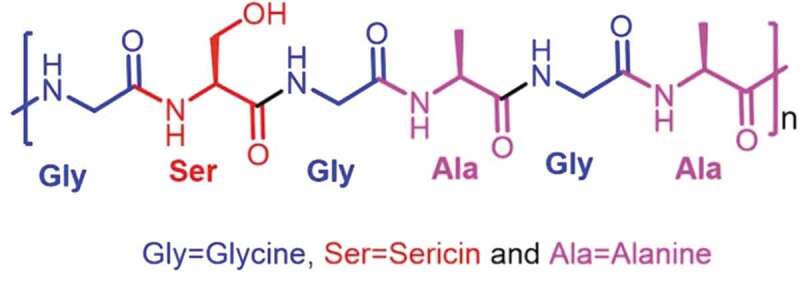
Structure of silk fibroin [18]

SF shows distinctive properties such as biocompatibility, unchallenging chemical modification, resistance to degradation *in vivo*, and its assembly into various material formats from aqueous to non-aqueous solvents, due to its various functional groups at the side and terminal positions of its structure (Figure 2) [17].

### 2.1. Sources of SF

There are different sources of silk, such as scorpions, mites, bees, silkworm, and spiders, which have SF in their glands [19]. The mostly used are arthropods, silkworms, cocoons and spiders or spider webs.

#### 2.1.1. *Cocoon (*Bombyx mori*) silk*

SF of *Bombyx mori* has a diameter of 10 to 25 μm and comprises two protein chains with different number-average molar masses: one is a shorter chain with number-average molar mass around 26 kDa and the other is longer around 390 kDa. Both of them are covalently bonded through disulphide linker between Cys-20 from long chain and Cys-172 from short chain, having 20^th^ and 172^nd^ residues of carboxyl terminus, respectively [20]. Moreover, both chains are in equal number and also noncovalently connected with glycoprotein [21].

#### 2.1.2. *Spider (*Nephila clavipes*) silk*

Silks obtained from different spider species have distinct chemical structures and characteristics. For instance, silk from *Kukulcania hibernalis* shows the highest stiffness of 22 ± 13 GPa, from *Leucauge venusta* has a maximum strength of 1470 ± 263 MPa, while that from *Scytodes sp*. has the highest toughness of 230 ± 85 GPa. Their varying mechanical properties are probably due to the difference in silk collection method and/or in species to species. For example, silk from *Kukulcania hibernalis* is collected when they are walking, from *Leucauge venusta* is gained when spiders are allowed to place themselves on a dragline from a raised platform, and from *Scytodes sp* is obtained by forcible silking [22]. In addition, silk from *Nephila clavipes* has two types of proteins: ampullate spidrions protein 1 and 2 (Ma Sp1 and Ma Sp2) [23]. On the basis of gel electrophoresis, major ampullate has molar mass around 275 kDa, whereas its molar mass on the basis of size exclusion chromatography is 740 kDa and that of smaller ampullate is 290 kDa [24]. Existence of sericin is not observed.

#### 2.1.3. Structural difference between silks from cocoons and spider

The basic structures of silks from silkworm and spider are almost the same since both possess microfilament clusters (0.5–2 μm) which contain semi-crystalline domains except some differences at nano meter level about fundamental and anatomical features (Table 1) [25–27].

**Table 1.** Structural difference between cocoon’s and spider’s silks

No.	Features	Silk of cocoons	Silk of spider
1	Size	600-1500 m from single silkworm	137 m from ampullate gland of spider and 12 m from spider web
2	Major amino acids	Glycine, alanine, serine	Glycine, alanine, glutamic acid, proline, arginine
3	β-Sheet structure	Yes	Yes
4	Components of crystalline domain	Glx (x = alanine, serine, threonine, valine)	(Alanine or glycine) + GPGX (X = proline,glutamine)
5	Components of less crystalline domain	Fibroin heavy chain made up of 25 identical amino acids	GGX glycine helix

#### 2.1.4. Dominance of cocoon silk over spider silk

**Figure 3.**
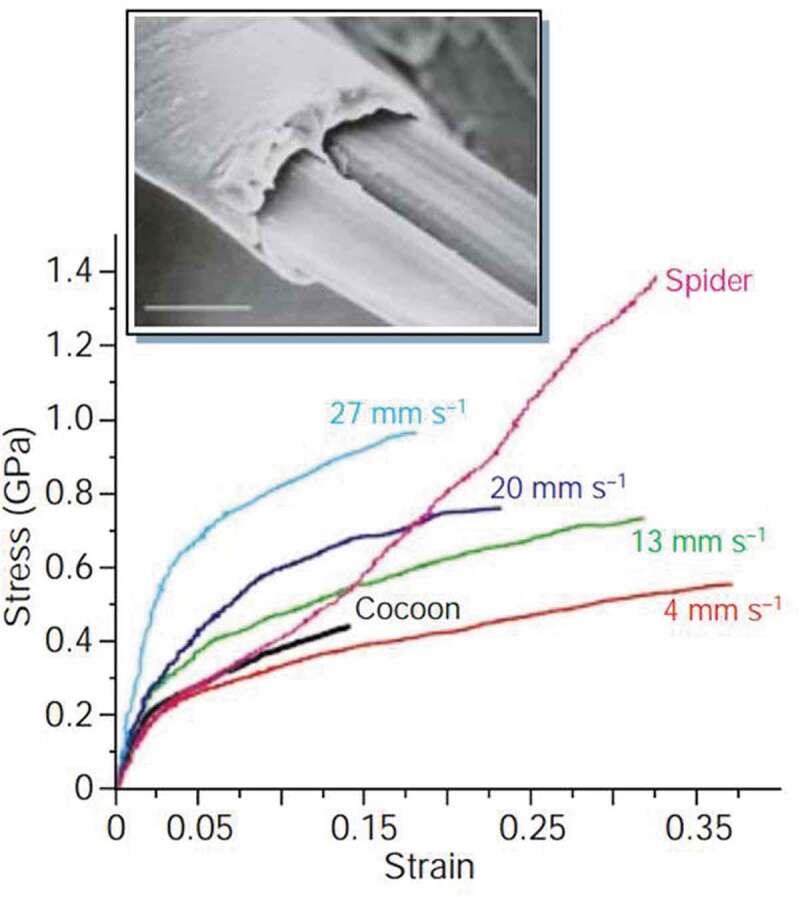
Mechanical strength comparison of silks obtained from the silkworm *Bombyx mori* drawn at different speeds [29]

As compared to spider dragline silk, silkworm silk is supposed to be much weaker in mechanical properties and less extensively commercialized, so that it has been hailed as a ‘super-fibre’. Under controlled conditions the mechanical properties of silkworm silk can approach to that of dragline silk. Thread quality of silkworm is better than that of spider silk by changing spinning habits, rather than by having their silk genes altered. Typical silkworm silk obtained from *Bombyx mori* cocoons has a tensile strength of about 0.5 GPa, a breaking energy (toughness) of 62.104 J g^−1^ and a breaking elongation of 15% [28]. On the other hand, *Nephila* spider dragline silk has strength of 1.3 GPa with a breaking elongation of 40%, and a toughness of 162.104 J g^−1^. The mechanical properties vary considerably for each other. Silkworm silk is traditionally obtained from a natural cocoon that is spun by the moving silkworm, which accelerates and decelerates its head in arcs attached at points that correspond to each change of direction. For spider silk, this variability is due to the spinning conditions, which are affected by the spider’s body temperature and the speed of drawing. Under steady and controlled conditions the artificial reeling of silk produce silk fibres, which are superior to naturally spun fibres. As a result, silkworms on one hand produce more brittle and stronger fibres at faster spinning speeds, but on the other hand slower spinning speed lead to weaker and more extensible fibres (Figure 3) [29].In addition, the mechanical strength of cocoon silk produced under certain conditions is approachable to that of spider silk. Besides, cocoons silk also shows some other advantages over spider silk. The details are given in Table 2.

**Table 2.** Dominance of cocoon silk over spider silk

NO.	Properties	*Bombyx mori* SF without sericin	Crosslinked collagen	Polylactic acid
1	Modulus (GPa)	15–17 [30]	0.0018–0.046 [31]	1.2–3.0 [32]
2	Ultimate tensile strength (MPa)	610–690 [30]	47–72 [31]	28-50 [32]
3	% strain at break	4–16 [30]	12–16 [31]	2-16 [32]
4	Water contact angle (°)	—	84.2 ± 0.8° [33]	—

### 2.2. Silk versus other biomaterials

Although many biomaterials are being used in bone regeneration, SF has several advantages over the others, which are allogeneic or xenogeneic in origin. For instance, SF is comparatively inexpensive because of its alkali- or enzyme-based degumming procedures as compared to other biomaterials, which require special isolation and purification methods. Only removal of sericin covered at the outer layer of SF is needed, which makes its processing much easier. Another important factor is that SF is economically advantageous because of large processing infrastructure. SF causes less inflammatory reaction [34] and has a slower degradation rate than many other biomaterials [35,36], though it is considered as non-degradable biomaterial according to US Pharmacopeia’s definition. However, it is actually enzymatically degradable since proteolytic enzymes are responsible for its degradation [35]. This enzymatic degradation involves two steps: the first step is the adsorption of silk by enzymes, and the second is silk digest by enzymes. As a result, corresponding amino acids are produced and thus absorbed *in vivo*. On the other hand, in the degradation process of other biomaterials, such as polyglycolides and polylactides, acidic products are released in the host body that can cause serious health problems. Besides, SF also shows good biocompatibility with the body tissues and retains its mechanical strength over a long time because of its nanocrystalline structure (Table 3) [37,38].

**Table 3.** Comparison of SF with other biomaterials

No.	Points of dominance	Cocoon silk	Spider silk [28]
1	Size	600–1500 m from single silkworm	137 m from ampullate gland of spider and 12 m from spider web
2	Nature of structure	Homogeneous	Heterogeneous
3	Domestication	High	Less
4	Productivity	High	Less
5	Processing	Easy	Difficult

### 2.3. Preparation of SF

#### 2.3.1. Method 1

Cocoons of *Bombyx mori* are degummed in 0.02 M aqueous solution of Na_2_CO_3_ at 100°C for 30 min and then rinsed with distilled water to remove sericin. SF is dried before adding in a solution of CaCl_2_/CH_3_CH_2_OH/H_2_O with molar ratio of 1:2:8. By dialysing for 96 h using cellulose membrane along with distilled water, SF sponges are obtained with water-content changes after every 24 h [39,40].

#### 2.3.2. Method 2

**Figure 4.**
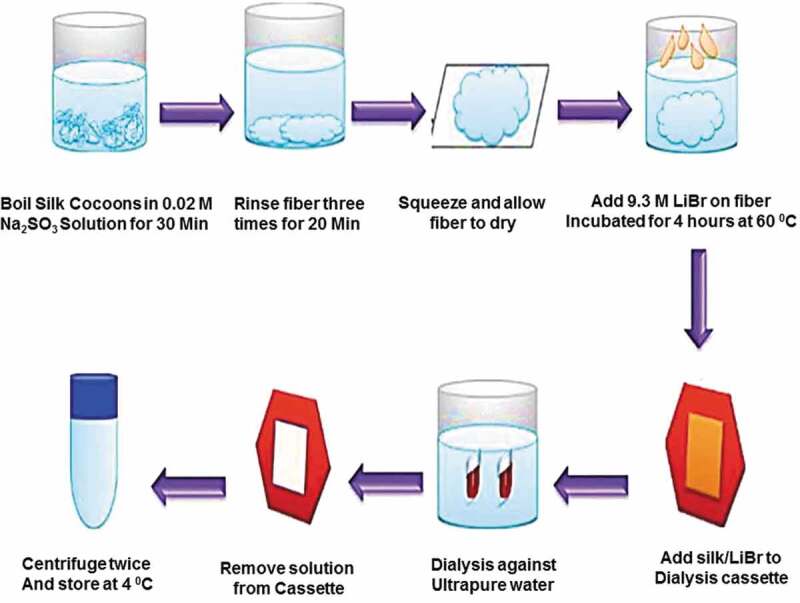
Schematic representation of extraction of SF from cocoons [41]

In a 0.02 M solution of Na_2_CO_3_ cocoons are boiled for 45 min and washed with distilled water to remove small proteins. To make 20% w/v solution, SF which is dried at 40°C for 24 h, is added in 9.3 M LiBr solution that is prepared at 60°C for 3 h. Afterwards, it is dialysed for 3 days and freeze dried to get purified SF, which is used to form solution of any required strength. Dialysed SF solution is centrifuged to remove the solid particles, the obtained clear solution can be used as it is or it can be lyophilized to get solid material. A 17% w/v SF solution with hexaﬂuoroisopropanol (HFIP) is prepared by the dissolution of the lyophilized SF at normal temperature for 24 h before experiment (Figure 4) [41].

### 2.4. Controlled morphology of silk biomaterials

**Figure 5.**
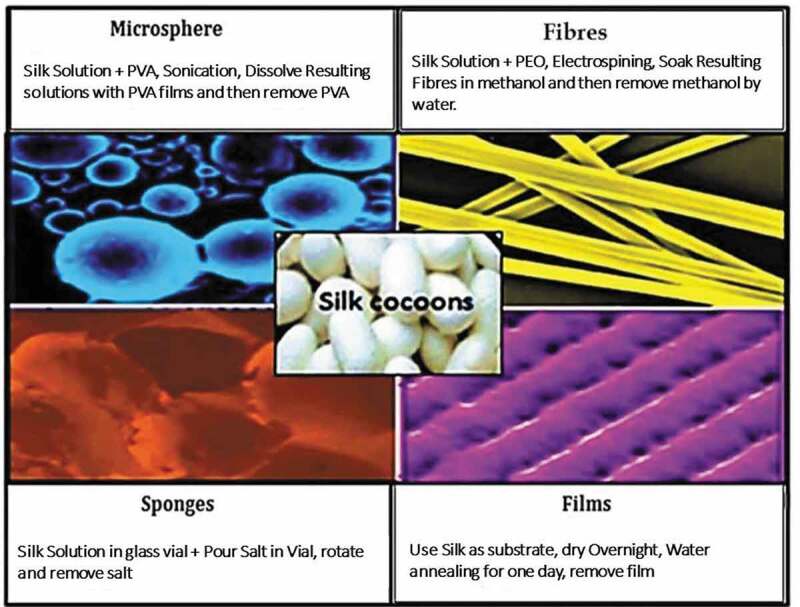
Schematic representation of materials fabricated from silk fibroin: 4 days are required for complete extraction of silk fibroin, and different materials can be obtained at different intervals [41]

For clinical application, silk fibre solutions are used to develop different morphological materials followed by reprocessing (Figure 5).

#### 2.4.1. Silk fibres

Silk fibres are formed by reeling from cocoons and utilized both in gumming (virgin) and degumming (black braided silk) forms. Silk fibres have various diameters (nanometres to micrometres) depending on the method used for their preparation [23]. Electrospun silk fibres show uniform size with diameter almost 0.8 μm and modulus of elasticity of about 13.6 GPa, which is much less than native silk fibre due to harsh processing conditions adapted for reconstituted silk. Moreover, these conditions can remove the key functional and structural aspects of natural silk [42].

#### 2.4.2. Silk sponges

Silk sponges have several applications in tissue engineering, such as disease modelling and implant materials like femur and mandibular defect, due to their 3D porous structure. Silk sponges are either aqueous or organic (e.g. HFIP) based. Water-based sponges have a high rate of degradation and better pore connectivity than HFIP-based ones. In contrast, HFIP-based sponges have stronger mechanical properties and smoother surface.

#### 2.4.3. Silk microspheres

Silk microspheres are used as therapeutic materials and carriers for growth factor. SF microspheres have been synthesized by a variety of processes, e.g. spray drying, lipid template, salting-out, freeze induced self-assembly, water-in-oil emulsification, casting, dissolution of polyvinyl alcohol (PVA)-silk blend films, and laminar jet break-up of an aqueous silk solution. Although SF nanoparticles have been fabricated successfully, they are less well suited to depot applications since their small size and high surface area-to-volume ratio leads to a rapid drug release [43]. In one of these procedures, a non-saturated fatty acid, 1,2-dioleoyl-sn-glycero-3-phosphocholine, is used with a concerning molecule to enclose water based silk microsphere. Microspheres formed by this method are approximately 2 μm in diameter. In another method, silk is incorporated with PVA to make microspheres. This yields microspheres with a size ranging from 300 nm to 20 μm. Moreover, size can be controlled by controlling the amount of silk in solution of PVA [41].

#### 2.4.4 Silk films

Silk films are tested in the adhesive property with pattern cells, degradation, and the possibility for release and screening of materials *in vitro* and *in vivo* environments. Water-based processing of SF favours incorporation of bioactive molecules with it. Silk films may be well patterned or non-patterned. Film thickness can be changed by varying the quantity of silk solution used. To increase the pore density, silk solutions are mixed with polyethylene oxide [41]. Silk scaffolds find different medical applications because of their different morphologies as shown in Table 4 [20,44–56].

**Table 4.** Clinical applications of silk scaffolds

Applications	Type of tissue	Scaffold format
Tissue engineering	Bones	Sponges (both HFIP and aqueous)
		Fibres
	Cartilage	Sponges (both types)
		Fibres
	Soft tissue	Sponges
	Vascular tissues	Fibres
	Cervical tissues	Aqueous sponges
	Cornea	Films
	Skin	Fibres
Drug delivery	Drug delivery	Microspheres
	Growth factor delivery	Microspheres
	Small molecules	Microspheres
Implant material	Anterior cruciate ligament (ACL)	Fibres
	Femur defects	HFIP sponges
	Mandibular defects	Aqueous sponges
Disease modelling	Breast cancer	HFIP sponges
	Autosomal dominant polycystic kidney disease	Aqueous sponges

## 3. Hydroxyapatite

The remarkable osteoconductivity, proliferation, osteointegration, biocompatibility and bioactivity of HA make itself a most promising candidate material for bone regeneration [57]. Ca_10_(PO_4_)_6_(OH)_2_ is its general molecular formula which is a member of apatite family and forms non-organic portion of bone [58]. It is used in bone treatment, drug release, as well as dental implants [59].

### 3.1. Structure of HA

**Figure 6.**
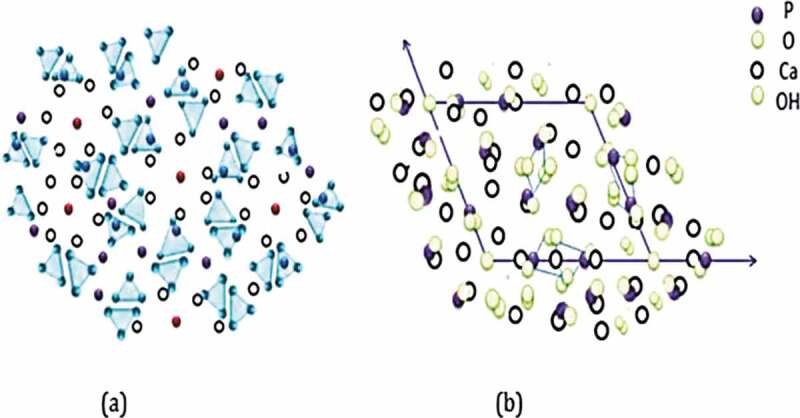
(a) atomic structure of HA and (b) its projection along c-axis [60]

HA has hexagonal structure with the calcium-to-phosphate molar ratio to be 1.67, and it has 44 atoms per unit. This arrangement of atoms plays a key role in its usage as the engineering material of bone tissue. Atomic structure and its projection along the axis can be visualized in Figure 6, where phosphorus, oxygen, calcium and hydroxyl ions/atoms are arranged in an ordered manner.

### 3.2. Size effectiveness

HA is produced both in micro- and in nano-sized particles but many recent studies have shown better osteoconductivity property in case of nano-sized hydroxyapatite (nHA) because of its size resemblance to natural HA found in bone [61]. nHA also shows improved protein adsorption and osteoblast adhesion in comparison with micron sized HA [57,62]. Moreover, nHA shows nontoxic behaviour towards mesenchymal cells [63].

### 3.3. Natural sources of HA

#### 3.3.1. HA derivation from mammalian bones

The size distribution, properties, efficiency, and phase purity of HA obtained from natural precursors, mainly from mammalian bones, depend on various factors such as temperature of calcination, extraction technique, and bone type. Bones that are most commonly used for the extraction of HA are bovine bones. Animal bones are first washed with pure water, boiled, washed with *aq*. NaOH or hypochlorite solution to remove proteins and dirt, and then dried. To control morphology and size of the final product, bones are then treated in two ways. One is to cut them into small pieces, and the other to mill them for times to powders. The processed bones are then heated from 600 to 1400°C for calcination so that all organic matters are cleared away. Selection of calcination regime is very important. It should avoid thermal decomposition but insure to destroy pathogens that could be responsible for disease transfer from cattle to person [64]. HA obtained from different mammalian sources may have slightly different properties (Table 5). It might be due to the difference in the origin of source or variable temperatures and methods that are applied for its extraction.

**Table 5.** Characteristics of hydroxyapatite obtained from mammalian sources

No.	Source(animal+ extraction method)	Calcium/phosphorus ratio	Shape	Calcination (T/°C)
1	Bovine, sheep and hen femur bone +sheep skull.	1.46–2.01	Irregular	600-1100 [65]
2	Defatted pork bone pulp	—	—	650-950 [66]
3	Cow bone by (a) hydrothermal hydrolysis (b) subcritical H_2_O extraction (c) thermal decomposition	1.52–1.9	Nanorods	[67] 250 275 750
4	Cow bone	1.9	—	1000 [68]
5	Defatted bovine bone	1.7	Needle shaped	800 [69]
6	Cow bone through thermal and mechanochemical way	—	Spheroidal and polygonal	800 [70]
7	Bovine bone through transferred arc plasma	1.93	—	[71]
8	Human, pig and porcine bones	—	—	600-1200 [72]
9	Bovine bones by ball milling	—	Spherical	800-1100 [73]
10	Cortical femoral bovine bone	—	Equiaxial	900 [74]
11	NaOH-treated bovine bone	>3	Interconnected with pores	900 [75]
12	Cow femur bone	Almost 1.6	Round	[76]

#### 3.3.2. Marine/river sources

About 50% of fish utilization is provided by marine/river captured fisheries, which produces Ca- and HA-rich waste. Fish bones containing excessive amount of HA are treated with hot water and sometimes with various alkaline solutions, to get rid of all organic impurities, followed by calcination at a high temperature to obtain pure HA [64]. Properties of HA obtained from different fish sources are shown in Table 6.

**Table 6.** Properties of HA obtained from fish sources

No	Source	Ca/P ratio	Shape	Calcination (°C)
1	Big eye tuna bone	1.76	Rod like	900 [77]
2	Pseudoplatystoma corruscans, Paulicea bones	1.64	Rod like	900 [78]
3	*Sepia officinalis* bones	1.64	Rod	900 [79]
4	*Thunnus thynnus* and *Xiphias gladius* sword bones	~1.9	Rod	600 and 950 [80]
5	*Oreochromis niloticus* scales	1.7	Hexagonal	950 [81]
6	*Nile tilapia* scales	1.8	Occasionally circular	950 [82]
7	*Gadus morhua* bone	1.5	Needle like	900-1200 [83]

#### 3.3.3. Plant sources

HA is applicable as bone scaffold template due to its high strength, toughness, and stiffness like wood. HA can be prepared from CaCO_3_, algae and corals due to its porosity and interconnectivity. For example, Biphasic HA can be made from red algae [84], and *Corallina officinalis* is the best option for HA extraction as it contains calcium carbonate. Hydrothermal method can be used to convert calcite from *Phymatolithon calcareum* (red algae) into HA [85]. Additionally, several studies showed that *C. edulis* (khat), trifolium, mint, green tea and basil can be used for the preparation of HA [86].

#### 3.3.4. Biogenic sources

Sintering temperature, sintering duration, and composition of ceramic materials are the factors that can control the mechanical properties. In this aspect, eggshells and seashells are best options to produce high quality biomaterials because of their close resemblance to human hard tissue. Carbonated nHA is the main inorganic component of teeth and bones [87,88]. Molluscs’ shells can also be converted to HA powder by *in vitro* treatment at room temperature [89].

A large number of eggshells are produced every year from hatcheries, houses, restaurants, and bakeries as a waste. Similarly, a large amount of seashells and other calcite materials are also abundant resources. About 94% calcium carbonate, 1% magnesium carbonate, 1% calcium phosphate, and 4% organic substances are present in an eggshell [90]. As a low cast and readily available material, eggshells can be a thorough source of calcium that is used to transform to HA. Table 7 is illustrating the properties of HA obtained from eggshells and seashells by different methods.

**Table 7.** Properties of HA from biogenic sources

No.	Source	Ca/P ratio	Particle size (nm)	Morphology	Secondary phase	Calcination (T/°C)
1	Egg shell	1.65	—	—	β-TCP	900 [91]
2	Coral shells	—	—	—	—	— [91]
3	HCl+DAHP solution treated Egg shells	1.67	50	Rectangular	—	[92]
4	Hen Egg shells	>1.67	18	Spherulite	—	800-1200 [93]
5	Chicken Egg shell	—	~35	Prolate spheroidal	—	700 [94]
6	Hen Egg shell	1.63	—	—	—	[95]
7	Egg shells treated with *DAHP+EDTA		78	Flower like	—	[116]
8	Egg shell treated by precipitation technique	1.67	~35	Globule like	—	900 [96]
9	Oyster shell		—	Rod like	—	1000 [97]
10	Ground waste egg shell			Flower like	—	— [98]
11	Fruits and egg shell waste	1.57–1.77	12-49	Needle like and rod like	—	— [99]
12	Egg shell waste	2.20	—	Spheroidal	β-TCP	1100 [105]
13	Mussel shell	1.61	—	—	—	— [100]
14	Egg shell	—	—	—	—	600 [101]
15	Egg shell	—	15 k-35 k	Flower like	—	— [102]
16	Sea urchin shell	—	—	Rod like	—	900 [103]
17	Egg shell	–	60	Whiskers	CaHPO_4_	700 [104]

* DAHP =** **‘2,4-diamino-6-hydroxypyrimidine and EDTA = ethylenediaminetetraacetic acid’

### 3.4. Preparation of HA

Wet methods for HA preparation are most commonly used, such as aqueous solution, co-precipitation, precipitation by emulsion, and template and sol-gel technique. Reactant concentration, temperature, and pH of solutions are the factors which need special attention to control [105].

#### 3.4.1. Chemical precipitation

The most promising method for production of mesoporous HA is chemical precipitation. Various chemicals of calcium and phosphate sources, surfactants, and pH-controlling reagents are involved in this reaction. Commonly used source of Ca^2+^ is calcium nitrate tetrahydrate (Ca(NO_3_)_2_ · 4H_2_O) and that of PO_4_^3-^ is diammonium hydrogen phosphate ((NH_4_)_2_HPO_4_) or dipotassium hydrogen phosphate trihydrate (K_2_HPO_4_ · 3H_2_O). In this procedure, dropwise addition of a phosphate salt to calcium salt with continuous stirring is first carried out. The stoichiometric ratio of Ca to P is kept at 1.67. In the second step, aging of sample is carried out at a constant temperature, and the precipitation is then washed, filtered, dried, calcined, and finally grounded into a powder at the last step [106–108].

To check the particle shape, surface area, size, and pore characteristics at different temperatures and pH, various surfactants are used to lower the surface tension of liquid in which they are dissolved. Two types of surfactants are usually applied, namely, soft and hard surfactants. Soft surfactants are further divided into cationic, anionic and non-ionic types.

**Figure 7.**
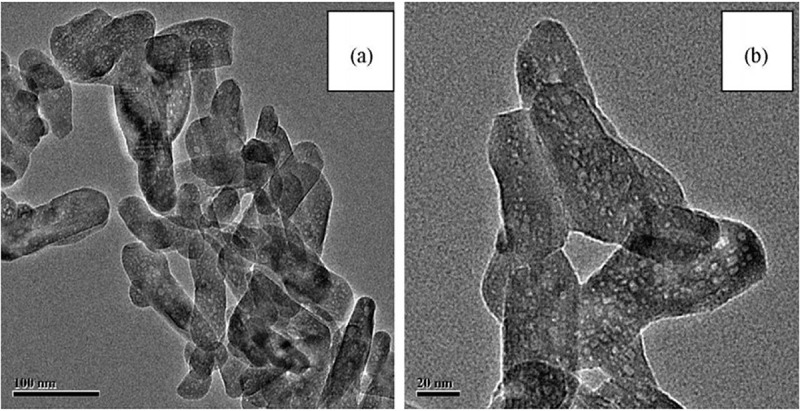
Typical transmission electron microscopy images of mesoporous HA calcined at 600 ° C: (a) 100 nm scale and (b) 20 nm scale [109]

Mesoporous carbon as the template with 2-dimensional hexagonal structure CMK-3 was used by *Xia* et al. for the preparation of rod like nanoparticles with 100 nm in length and 20 nm in width (Figure 7). Its pore size, surface area, and pore volume are 2.73 nm, 42.43 m^2^ g^−1^, and 0.12 cm^3^ g^−1^, respectively. Microbial test gives much better results with surface area of 86 m^2^ g^−1^ and pore width of 2–4 nm. Though these materials provide good results but drawbacks are also obvious. For example, method proposed by Xia et al. needs complex procedure to eliminate carbon that may cause serious experimental errors [109].

**Figure 8.**
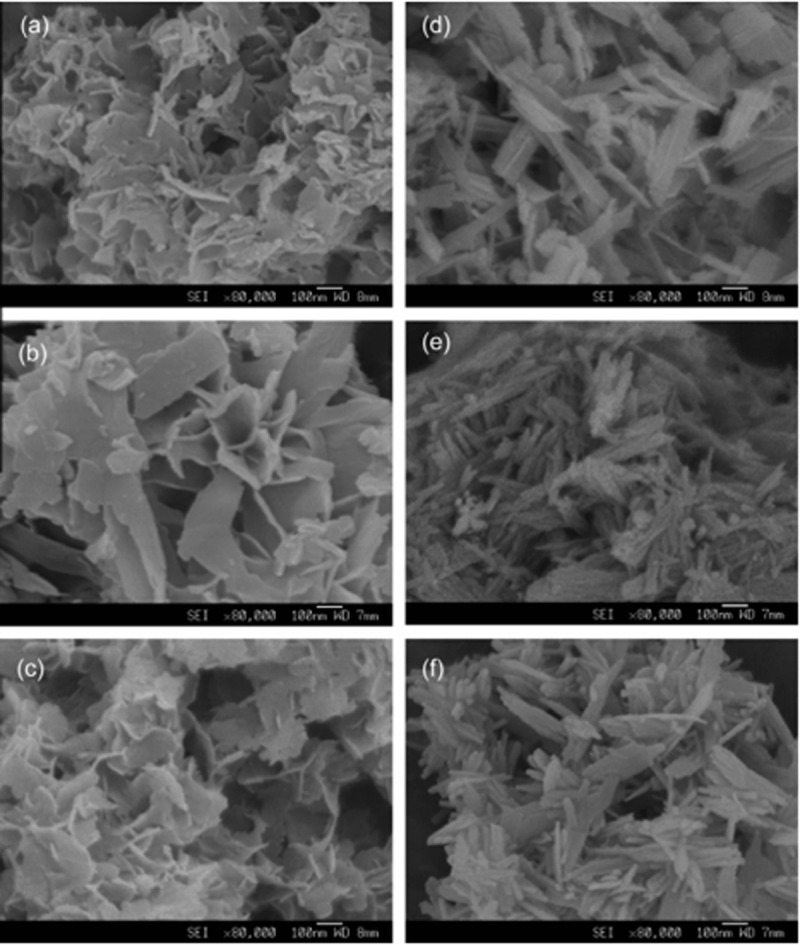
FE-SEM images of calcium phosphate nano-particles. (a–c) Nano-particles synthesized at 40 C: (a) 10% F127; (b) 40% F127; and (c) 80% F127. (d–f) Nano-particles synthesized at 100 C: (d) 10% F127; (e) 40% F127; and (f) 80% F127. Magnification is 80,000 times [108]

Compounds of alkyl trimethyl ammonium bromide are used as cationic surfactants, for example, cetyltrimethyl ammonium bromide (CTAB). By using CTAB, HA nanorods have been synthesized by Yao and his team with diameter of 50–100 nm and length of 500–1000 nm along with the pore volume of 0.0113 cm^3^ g^−1^ [110]. The size of these pores confirmed by nitrogen adsorption is 3 nm. Induction of pores in nanorods by CTAB is further confirmed by other studies [111]. By mixing cationic CTAB and anionic sodium dodecyl sulphate (SDS) surfactants, nanoparticles with different morphologies are prepared by Tari and co-workers [112]. The different shapes of nanoparticles depend on the concentrations of cationic and anionic surfactants. When cationic surfactant is higher in concentration, sheet-like arrangement of particles is observed, whereas high concentration of anionic surfactant leads to the formation of rod-like nanoparticles. It proves the morphology of nanoparticles can be controlled by the molar ratio between cationic and anionic surfactants. However, nanoparticles obtained in this way are of low surface area and their pore size is not uniform. To overcome these problems, non-ionic surfactants, including poly(ethylene oxide)-based triblock co-polymers, i.e. F-127 and P-123, and Tween-16, are used [108,113]. High concentration of F-127 (0.1 g mL^−1^) results in spherical nanoparticles with a diameter of 100 nm and a pore size of 5.8 nm, whereas low concentration (0.03 g mL^−1^) yields rod-shaped particles with diameters of 40–50 nm, lengths of 100–300 nm, and pore sizes of 2.5–3 nm (Figure 8). There results once again confirm the dependence of morphology of nanoparticles on the concentration of surfactant.

In order to get particles of HA with larger pore size, the use of a mixture of P-123 and Tween-16 is tried, which results in hollow nano-spheres and nano-rods [113]. Citric acid addition to P-123 solution gives nanotubes. Nanospheres have a diameter of 60 nm and a pore size of 36 nm with a pore volume of 0.47 cm^3^ g^−1^, while nano-rods have a diameter of 35 nm and a length of 50–250 nm with pore volume of 0.34 cm^3^ g^−1^ and pore size of 15.6 nm.

#### 3.4.2. Hydrothermal

Highly crystalline HA nanoparticles are obtained by hydrothermal method, which has basic similarity with chemical precipitation method. The difference between precipitation and hydrothermal method is aging. The aging in hydrothermal process is carried out in an autoclave at higher temperature than boiling point of water [114]. High temperature in hydrothermal process improvises the phase purity and Ca/P ratio [115]. Improvements can be achieved in pore size and surface area of pores by using CTAB with F-127, F-87, and P-123, which are most commonly used surfactants in this method [115]. A closed packing of HA is achieved owing to the high temperature and concomitant high crystallinity. Stoichiometric ratio of Ca/P and crystallinity in HA make the hydrothermal method more promising in the future [105].

#### 3.4.3. Emulsion

Mesoporous HA is also prepared by emulsion process. HA nanoparticles with different morphologies, such as needle-like, spherical, and rod-shaped, have been synthesised by Kumar et al. by changing the condition of emulsion reaction between calcium nitrate tetrahydrate (Ca(NO_3_)_2_ · 4H_2_O) and phosphoric acid (H_3_PO_4_) as the main precursors for Ca^2+^ and PO_4_^−3^ sources [116].

Jarudilokkul and his co-workers showed that surface area can be reduced by increasing calcination temperature and reaction between (sorbitan ester) Span 20 and (ethoxylated spans) Tween 80 [117]. But if the temperature is kept low there will be a chance of impurity in the sample. Therefore, an optimum temperature is maintained at which polymeric surfactants can work best.

## 4. Silk fibroin/hydroxyapatite

SF is incorporated with HA to enhance the clinical application of HA due to its less inflammatory reactions [118,119].

### 4.1. Preparation

Different strategies are used to prepare SF/HA scaffolds, for example, electrospinning [120], freeze gelation and freeze drying [121]. In general, a commonly used method is freeze drying. Different steps involved in this process are as follows.

#### 4.1.1. Step 1

Main components of silkworm *Bombyx mori* are sericin (outer covering) and fibroin (inner brins). Outer covering sericin is removed by degumming process and fibroin is obtained from methods, as mentioned previously.

#### 4.1.2. Step 2

The starting materials for the production of nHA are CaCl_2_ and Na_2_HPO_4_. 400 mL (0.12 M) of Na_2_HPO_4_ aqueous solution is slowly added to 400 mL of (0.20 M) of CaCl_2_ aqueous solution. 0.02 g of citric acid is then added as dispersant. NaOH aqueous solution is used to maintain the pH at 11. Mixture is then stirred continuously for 4 h and afterwards aged for 3 h. Precipitates are collected by centrifugation, neutralized by ethanol and distilled water for 3 h, and ultrasonically treated at 80°C. Sample is then vacuum-dried at 60°C for 24 h.

#### 4.1.3. Step 3

**Figure 9.**
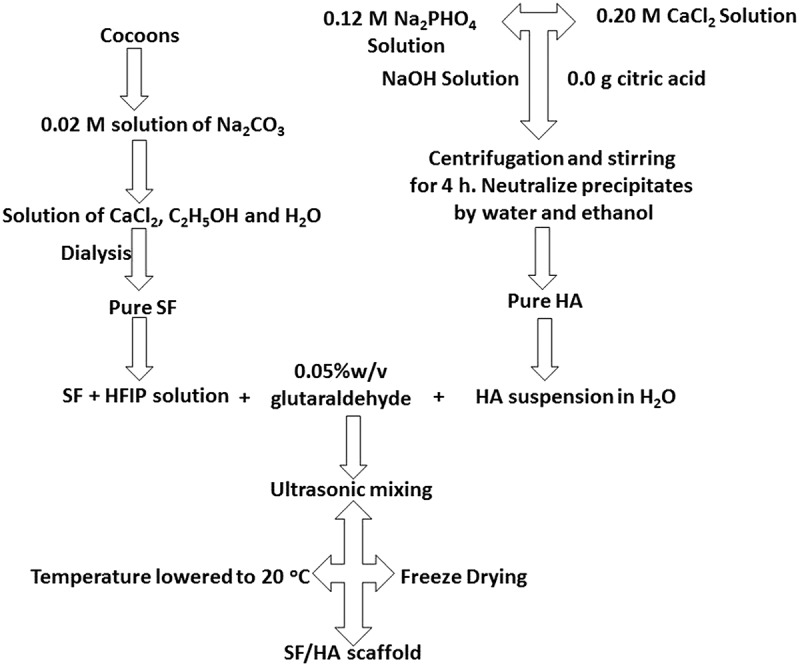
Schematic representation of preparation of SF/HA

HA solution is prepared by ultrasonication for 30 minutes in distilled water. Various amounts of SF solution are then added into different HA solutions. During mixing ultrasonication is still applied for uniform distribution of HA into SF constituent at a constant pH of 8. Glutaraldehyde (0.05% w/v) is added as a crosslinking agent and the mixture is then poured into a plate with a diameter of 23 mm and a depth of 18 mm. Temperature is lowered to −20°C and kept for 12 h, followed by freeze drying. In this way, SF/HA composites with different concentrations of HA can be prepared. Figure 9 illustrates the formation of SF/HA.

### 4.2. Preference of SF/HA over other HA based biomaterials

**Figure 10.**
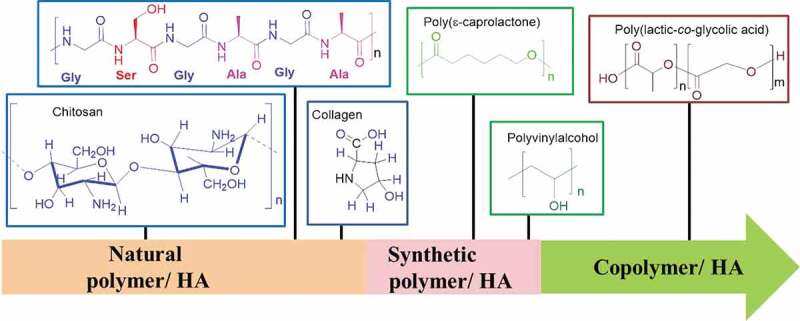
Types of polymers used in HA/polymer scaffolds

SF/HA shows better environmental stability, options for genetic control to tailor sequence, biocompatibility, mechanical properties, and ability to take the place of hard tissue material, making itself much more promising material for bone regeneration. Another important factor, which makes SF/HA superior to other biomaterials, is its advantageous porous structures that provide better transportation of blood and body fluids for metabolism and growth of bone. All these properties of SF/HA scaffolds guarantee its close resemblance to the natural bones. HA-based composite materials used in tissue engineering are presented in Table 8 and Figure 10, and discussed as follows:

**Table 8.** HA-based biomaterials in bone regeneration

No.	Composite	Method of preparation	Advantages	Disadvantages
1	HA-Chitosan	In situ chemical synthesis, freeze drying, co-precipitation	Pore forming ability, good binding capacity, anti-bacterial and biodegradable	Mechanical properties need to be improved [122–134]
2	HA-Collagen	Supercritical ﬂuid assisted process, cryogelation technique	Better cell attachment, proliferation and differentiation ability	Presence of charged and polar groups which critically affect the nucleation of the HA crystals on collagen membrane through chemical interaction [136–153]
3	HA-Polycaprolactone (HA/PLC)	Modified rapid-prototyping for nHA/PLC, pressure quench nHA/PLC	Good biodegradation, mechanical strength, growth of MSCs and guide their osteogenic differentiation.	Difficulty in new bone tissue binding [2,152–167]
4	HA-Polyvinyl alcohol	Freeze/thaw, spray drying	Good hydrophilicity, excellent chemical stability, useful for bone tissue engineering and articular cartilage repair.	Need further in vitro and *in vivo* studies [8,9,168–177]
5	HA-Poly(lactic-*co*-glycolic) acid	Selective laser sintering, Electrospinning	Good compressive strength and modulus of elasticity	Smaller pore size [178–182]

1. nHA-chitosan nanocomposites
  Pore formation, biodegradation and antibacterial properties make chitosan favourable for bone tissue engineering. However, mechanical properties of chitosan-based composites are not sufficient and need to be improved [122–134].
2. nHA-collagen nanocomposites
  The main component of bone is collagen that shows good cell adhesion and proliferation activities. When composited with HA, collagen shows cell differentiation as well. Collagen contains several kinds of negatively charged, positively charged, and polar yet neutral groups, in which the negatively charged groups undergo chemical reactions that suppress the nucleation of HA on collagen [135–151].
3. nHA-polycaprolactone nanocomposites
  Polycaprolactone (PCL) has applications in bone regeneration due to its nice bioresorbability and inexpensiveness. However, it is not an effective bioactive material because bone tissue makes very loose bond with it. This problem is being resolved by making its composites with HA, but still needs more improvements [2,152–167].
4. nHA-poly(lactic-co-glycolic) acid nanocomposites
  Poly(lactic-*co*-glycolic) acid (PLGA) is a biodegradable copolymer and widely used biomaterial in medical implants due to good mechanical strength and modulus of elasticity. However, its small pore size makes it less favourable for bone regeneration [8,168–177].
5. nHA-polyvinyl alcohol nanocomposites
  Polyvinyl alcohol can be applied in bone tissue engineering because of its good hydrophilicity and chemical stability, but it’s *in vivo* or *in vitro* applications require further investigation [178–182].

### 4.3. Characterization of SF/HA Scaffold

#### 4.3.1. Morphology

**Figure 11.**
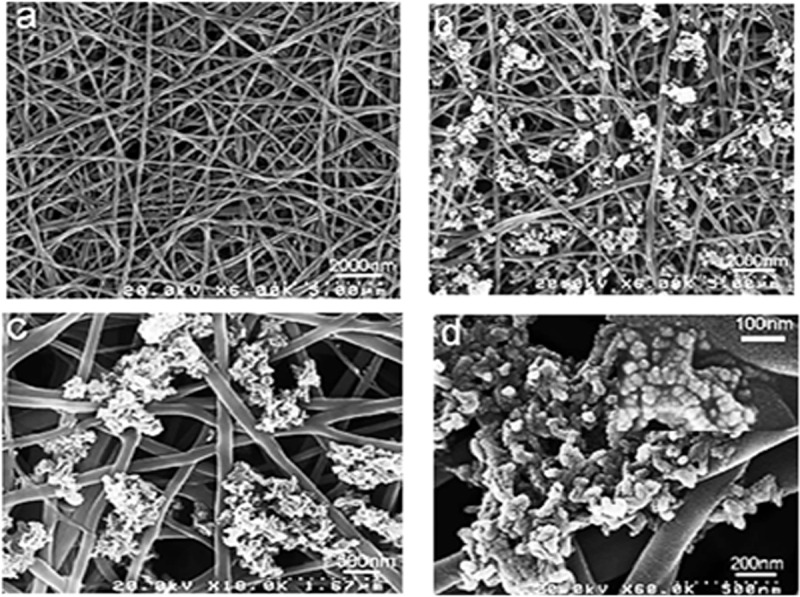
FE-SEM images of pure SF after three cycles of Ca-P treatment and mineralized SF/HA nanofibres: (a) pure silk nanofibres and (b–d) SF/HA nanofibres with different magnification [40]

SF nanofibres have diameters of 242 ± 34 nm, as shown in the field-emission scanning electron microscopy (FE-SEM) images (Figure 11). FE-SEM images also show that the deposition of HA on SF does not affect the macroscopic structure of SF. The deposited HA particles have nanocrystalline structures with diameters of 30–35 nm, as known from X-ray diffraction (XRD) analysis. With the concentration increasing, the roughness in the pore-wall also increases. HA nanoparticles are uniformly distributed on SF network and considered to be helpful in proliferation and cell adhesion due to biocompatible nature of HA.

#### 4.3.2. Secondary structure of SF/HA

##### 4.3.2.1. FTIR analysis

**Figure 12.**
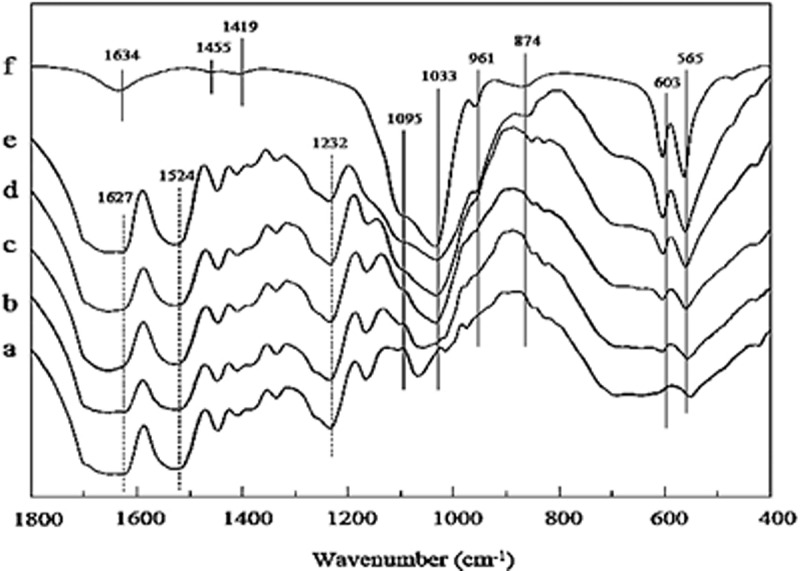
FTIR spectra of SF/HA porous scaffolds with different molar% contents of nHA: (a) 0%, (b) 10%, (c) 30%, (d) 60%, (e) 70%, and (f) 100% [184]

Fourier transform infrared (FTIR) spectroscopy has been extensively used to provide helpful information regarding functional groups present in different types of HA and SF based structures/composites. Specifically, FTIR analysis shows a band for phosphate group between 900 and 1100 cm^−1^ (P-O stretching) and another band between 500 and 600 cm^−1^ (P-O-P bending) [3]. The peaks at 1455 , 1419, 874 cm^−1^ are due to C-O stretching of dissolved CO_2_ from atmosphere in the solution [17,183]. Moreover, amide groups of SF show their absorption peaks at 1232, 1524 and 1627 cm^−1^ (Figure 12) [9]. For further detailed evidence along with FTIR investigation chemists still require different other characterization tools to explore and identify the different structures of SF and HA. Following figure presents a highly significant peak values to identify its structure.

#### 4.3.3. Crystal structure of SF/HA

##### 4.3.3.1. Energy dispersive X-ray spectroscopy

**Figure 13.**
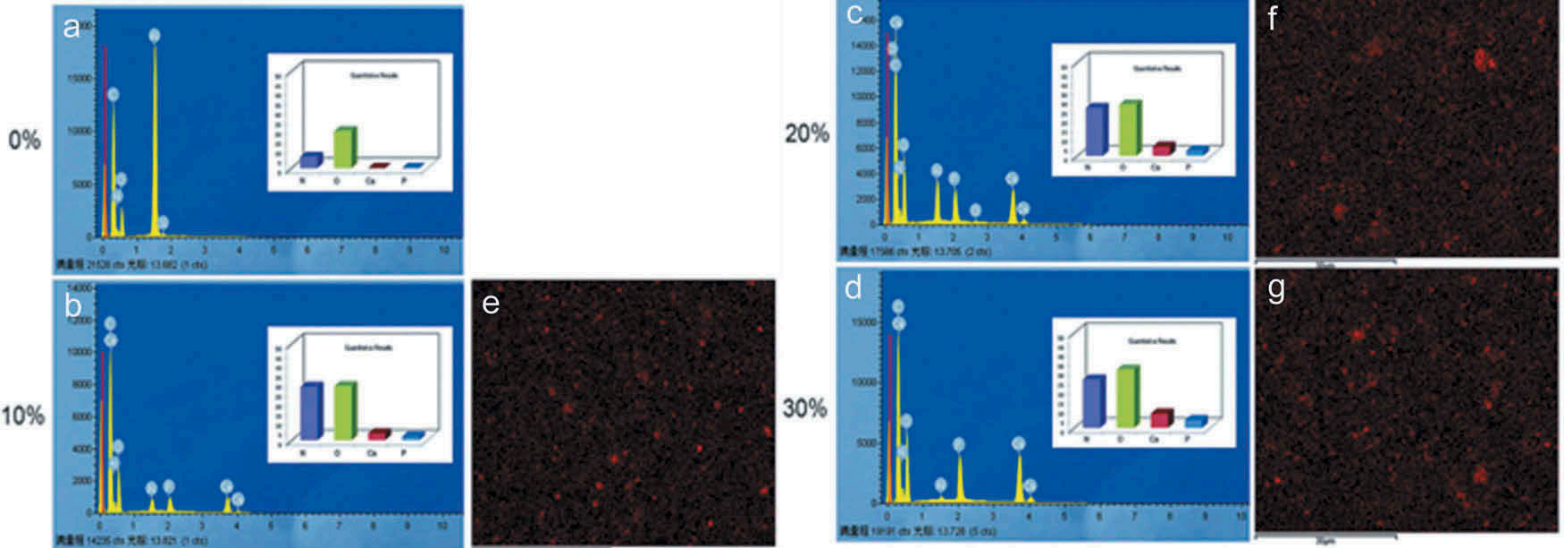
Elemental analysis of calcium (Ca), phosphorus (P), with oxygen (O) and nitrogen (N) in (a–d) Increase in SF/HA concentration is confirmed via EDS mapping of Ca (e–g) [185]

Along with atomic arrangement, the ratio of calcium and phosphorus is very important for understanding the mode of action of HA and SF. Elemental analysis of SF/HA by energy-dispersive spectroscopy (EDS) reveals incorporation of HA into SF scaffolds. Higher amount of Ca is observed as the concentration of HA increasing in scaffolds (Figure 13).

##### 4.3.3.2. XRD analysis

**Figure 14.**
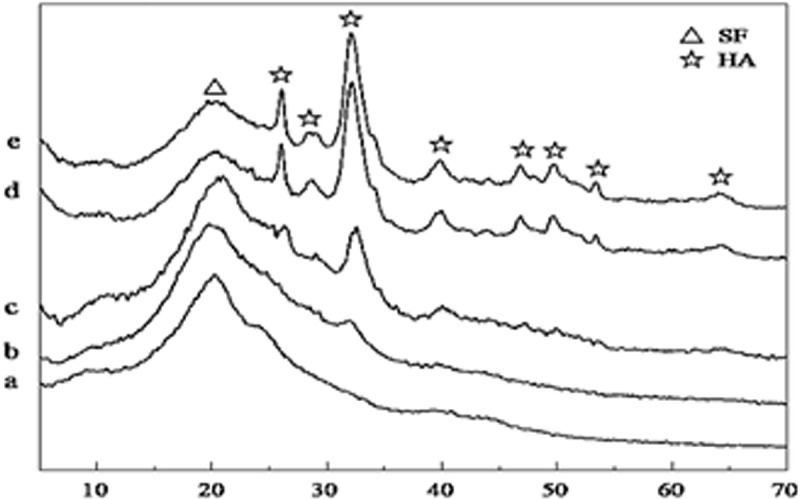
XRD patterns of different scaffolds of SF/HA composites with (a) HA0, (b) HA10, (c) HA30,(d) HA60, and (e) HA70 [184]. 2θ (degree)

Well-defined two peaks appeared in XRD patterns at 25.9° and 31.8° for Ca and P of HA and are identified as (002) and (211) peaks, respectively. Semicrystallinity in HA structure has been observed by broadening and overlapping of peaks (Figure 14) [1]. Another major peak appearing at 20.5° is attributed to β-sheet structure of SF. As concentration of HA increasing, the two peaks at 25.9° and 31.8° became more intense and anisotropic growth of HA is observed due to the existence of SF.

### 4.4. Properties of SF/HA scaffolds for bone regeneration

SF/HA scaffolds have various applications such as in bone transplant, drug delivery, growth factor delivery, and bioconjugates because of their biocompatible and osteoconductive properties. Major characteristics, which make SF/HA scaffold most compatible material for bone regeneration are discussed below.

#### 4.4.1. Mechanical properties of SF/HA

As the concentration of HA was increased from 0 to 30%, a slight change in the tensile strength was observed and which suggests that concentration of HA in scaffolds does not considerably affect the mechanical strength of SF/HA. It means the mechanical property of SF/HA decisively depends on SF.

#### 4.4.2. Hydrophilicity

**Figure 15.**
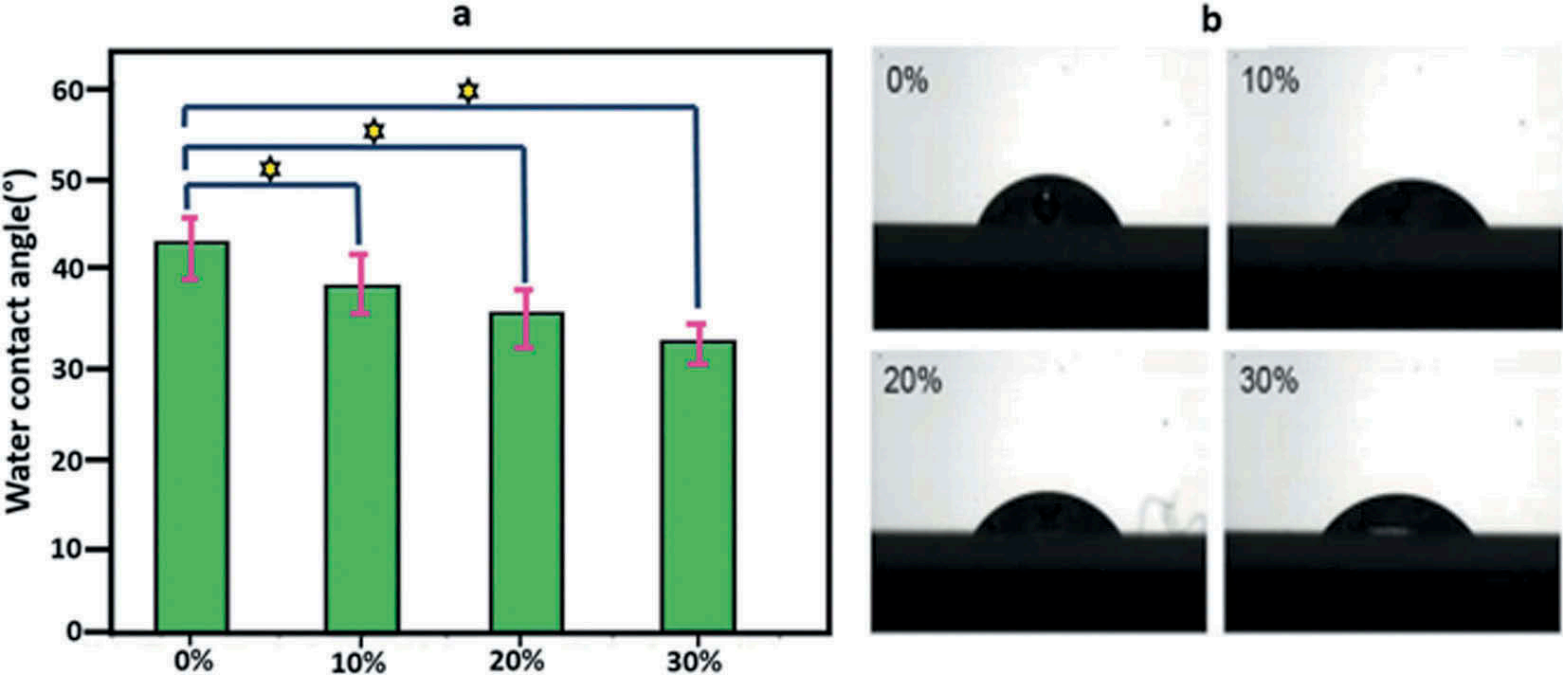
(a) Water contact angle of SF/HA scaffolds with different HA contents (p < 0.05) and (b) photographs of water droplets on SF/HA scaffolds [185]

The most important factor that affects the biocompatibility of any material is its hydrophilicity. By increasing HA content in SF/HA, the hydrophilicity of SF/HA increases in line with the decrease in the contact angle (Figure 15). Water drop on SF surface shows a contact angle of about 84°, whereas the optimum contact angle should locate between 55°and 75° for a suitable cell adhesive material [40]. Although SF with polar – OH and – COOH groups shows moderate affinity to water, its hydrophilicity should be further enhanced for biocompatibility by incorporating with HA bearing additional – OH and phosphate groups. These highly hydrophilic groups make the spreading of water droplets more favourable, which is in turn responsible for improved hydrophilicity. Hydrophilicity of SF/HA continues to decrease with the increase of the HA content, and SF/HA with around 30% HA content shows the highest hydrophilicity in the studied samples [185].

#### 4.4.3. Thermal properties

**Figure 16.**
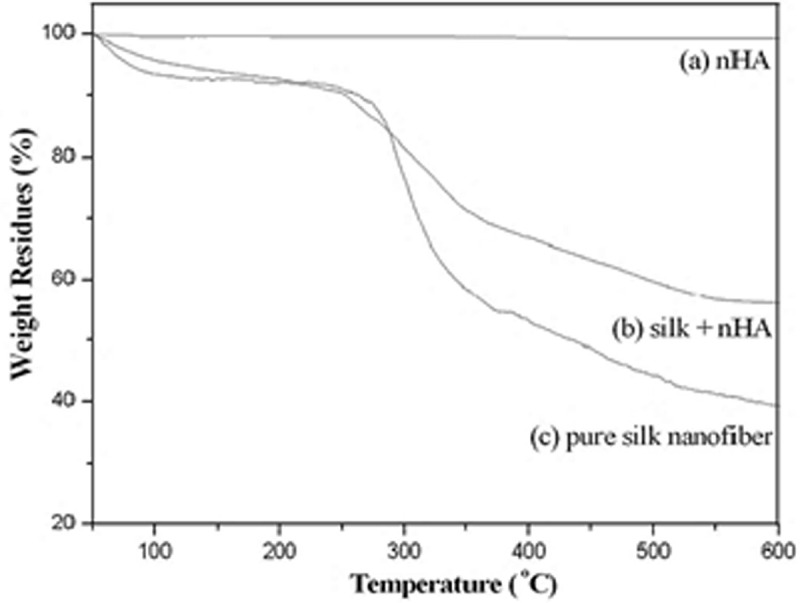
Thermogravimetric curves of nHA, pure SF and SF/HA (molar ratio = 8:2) [40]

nHA is thermally stable up to 600°C, as shown in Figure 16. For SF, a 7% decrease in the weight is observed due to the evaporation of water when temperature is changed from 90 to 105°C. In addition, a sharp decrease in weight is observed in a temperature range of 295–305°C due to its thermal degradation [57], and only 28.4% residues when temperature is raised up to 600°C. In contrast, SF/HA scaffold shows a similar thermogravimetric change within the scanning temperature. In the temperature range of 90–105°C, only 5% water loss was observed while 48.4% residue was remained at 600°C for the mineralized silk/nHA composite scaffolds [40].

#### 4.4.4. Cell proliferation

**Figure 17.**
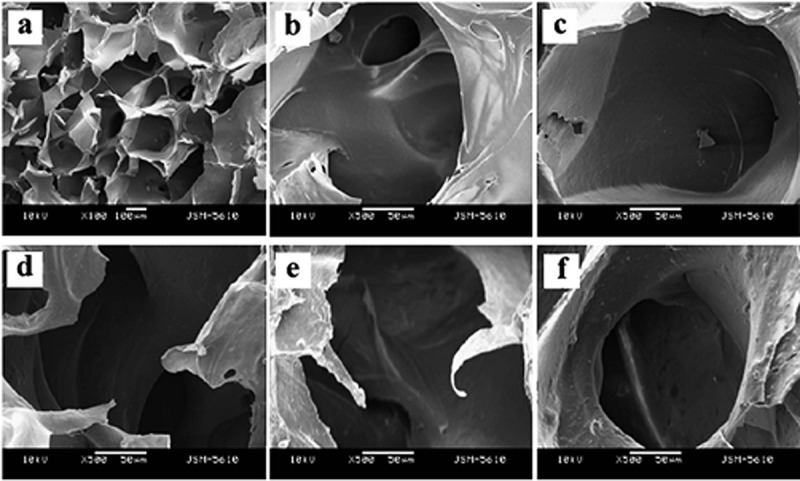
SEM images of porous SF/HA scaffolds with different molar contents of nHA: (a) 10% (b) 0% (c) 10% (d) 30% (e) 60% and (f) 70% [184]

The pore size of used materials plays a significant role in tissue regeneration. Various studies indicate that pore size of > 100–150 µm is desired for achieving better results in tissue growth. SF/HA scaffolds are of macroporous with the size range of 200–250 µm, as shown in Figure 17. Comparison of SEM images of SF and SF/HA shows no change in macroscopic morphology. However, the porous surface becomes rougher when content of nHA is increased, and the cell adhesion and proliferation activity of nHA will be enhanced. As for pore-wall surface, the microscopic wall becomes rougher by increasing content of nHA. The nHA particles are dispersed homogeneously throughout the SF network, and larger nHA aggregations are not detected. Moreover, for the cell adhesion and proliferation the nHA particles are inlaid on the pore-wall surface of composite due to the well-known biocompatibility of HA.

**Figure 18.**
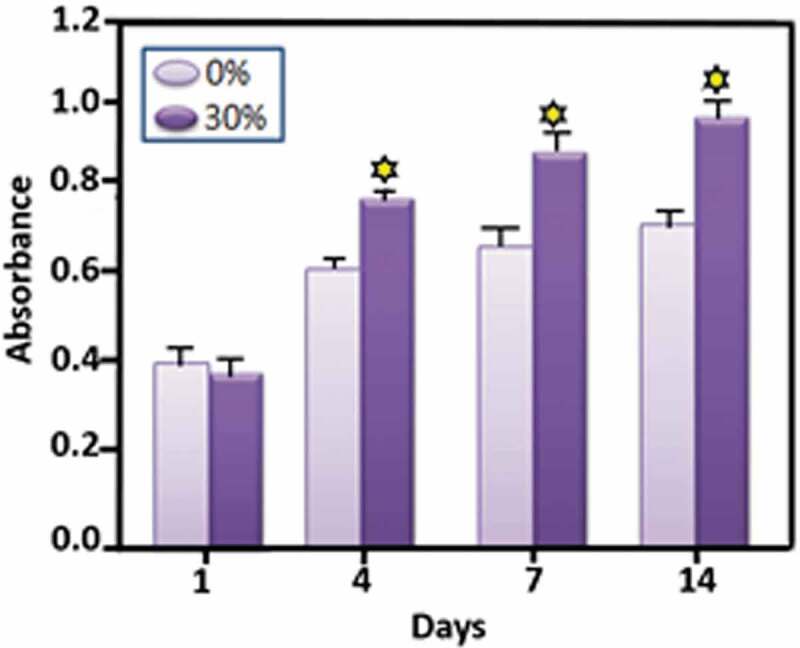
Cell proliferation investigation of rBMSCs cultured on 30% SF/HA (dark in colour) and pure HA (light in colour) [185]

**Figure 19.**
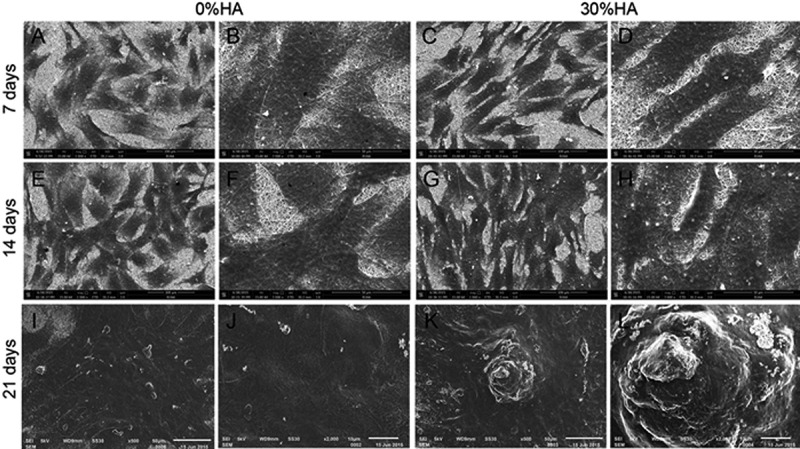
SEM images of attachment and proliferation of rBMSCs cultured on SF/HA scaffolds for 7, 14 and 21 days (a,c, e, g, i and k) scale bars = 100 µm; (b, d, f, h, j and l) scale bars = 50 µm [185]

The development and metabolic behaviour of cells cultured on the scaffolds is a special concern for clinical transplantation. Ding, et al. have studied the development and proliferation activity of rabbit mesenchymal stem cells (rBMSCs, a common osteogenic cells) on SF/HA scaffolds with different molar% contents of HA (0%, 10%, and 30% SF/HA, respectively). In this study, much higher metabolic rate of rBMSCs on 30% SF/HA has been observed as compared to that on pure HA after 4 days. The study concludes that SF/HA with higher HA content increases the proliferation activity of rBMSCs (Figure 18). SEM results also show higher cell adhesion and spreading of rBMSCs by 30% SF/HA (Figure 19). This result is in agreement with previous studies [185].

**Figure 20.**
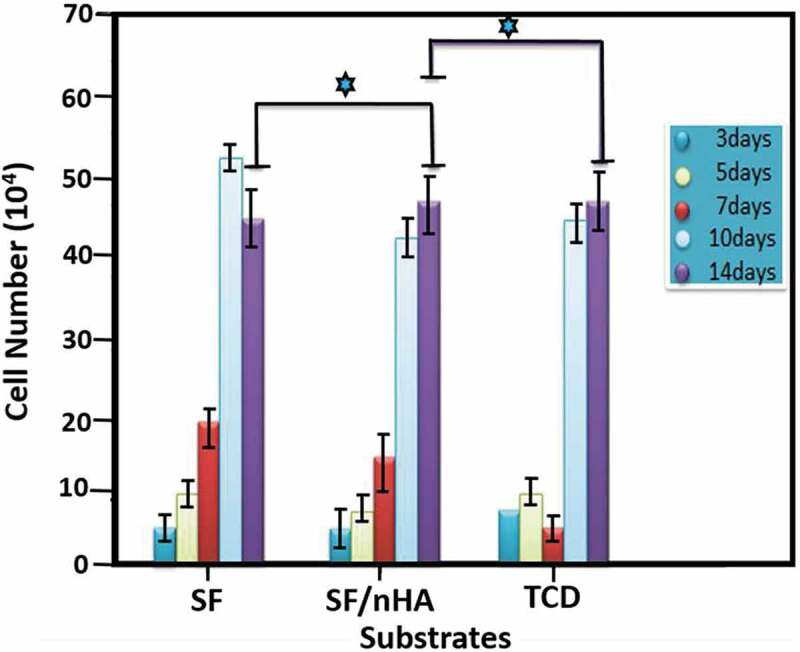
Proliferation activities of cells cultured on pure SF, SF/nHA, and tissue culture dish (TCD) from 3 to 14 days. (p ≥ 0.05) indicates significant increase in proliferation activity [40]

However, another study on the MC3T3-E1 cells which are osteoblast precursor cell line derived from Musculus (mouse) calvaria (skullcap) indicates an opposite behaviour (Figure 20). Kai and Wei et al. report that higher proliferation activity is observed on pure SF than on SF/HA even after a 7-day cultivation. According to their explanation, it may be due to the size, density and bulk distribution of nHA. Their study coincides with other studies in that surface morphology has significance influence on cell behaviour, as previously stated as curved surface of HA greatly reduces cell proliferation activity [40]. Nevertheless, this study also reveals that an increase of HA content has no negative effect on cell proliferation in latter stages of cell cultivation.

#### 4.4.5. Alkaline phosphate activity

Alkaline phosphate (ALP) is an enzyme found in different body tissues such as liver, kidney, intestine, and bone. It helps to break down various proteins. ALP test is very helpful in the diagnosis of bone problems like weakening, softening, and destruction. It also supplies information about vitamin D deficiency. ALP is an important part of bone matrix vesicle that breaks down the organic phosphate esters and forms apatite Ca-phosphate which helps in the initiation of cell differentiation process. ALP activity of rBMSCs and MC3TC-E1 cells suggests a remarkably enhanced cell differentiation with the increase of HA content. Moreover, a higher deposition level of Ca is observed on 30% SF/HA than on pure SF. As more Ca deposition implies higher degree of cell differentiation, bone formation ability will be increased by SF/HA scaffolds with higher HA content.

#### 4.4.6. Mineralization

**Figure 21.**
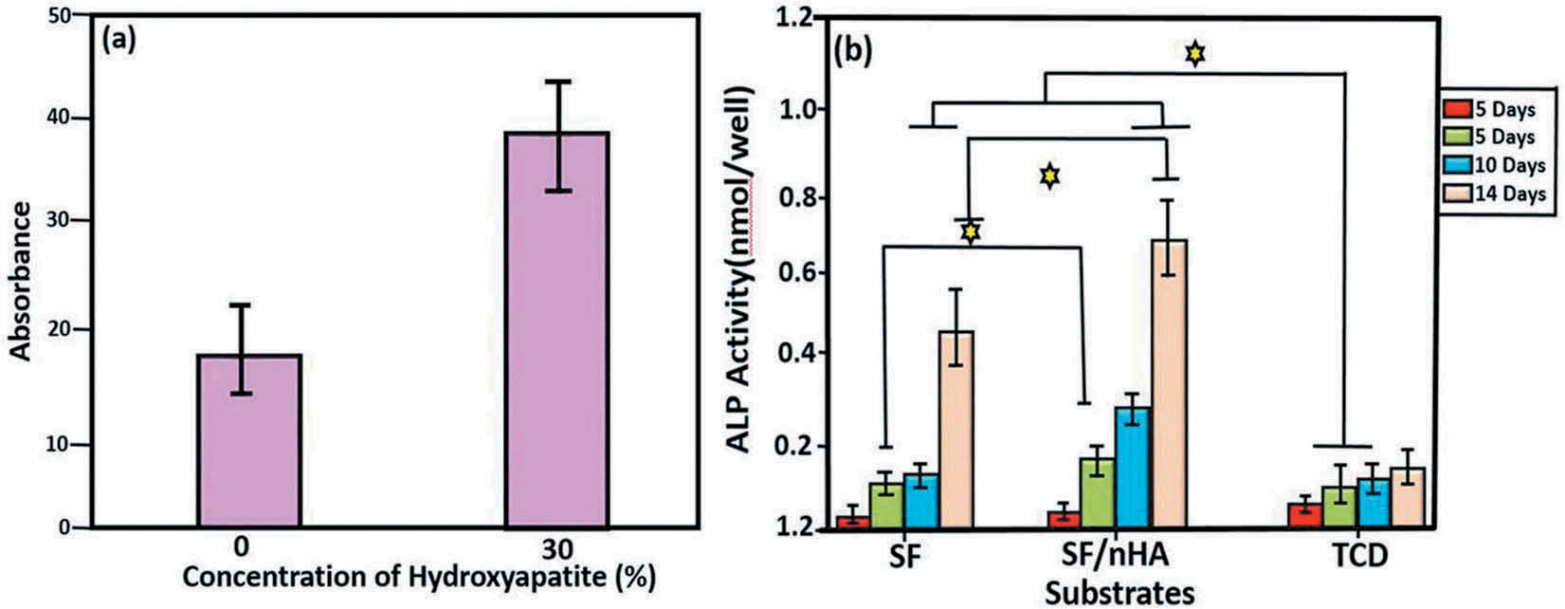
ALP activity. Osteogenic differentiation of (a) rBMSCs on SF/HA scaffolds with 0 and 30 mol% HA, and (b) MC3TC-E1 on pure and mineralized SF/nHA after 5, 7, 10 and 14 days. ⋆ shows difference in ALP activity [40,185]

The phase in which cells begins to release mineral matrix during osteogenic differentiation is called mineralization phase. It is typically determined by a dye (Alizarin Red S) that binds selectively to the calcium salts and hence can be used for mineral staining. The mineral deposition analysis of rBMSCs showed more quantity of Ca deposited on 30% SF/HA than pure SF in Figure 21. High mineral deposition ability means more mature osteoblast cells which shows more differentiation of rBMSCs and finally leads to deposit more extracellular cellular matrix.

### 4.5. SF/HA for bone regeneration

**Figure 22.**
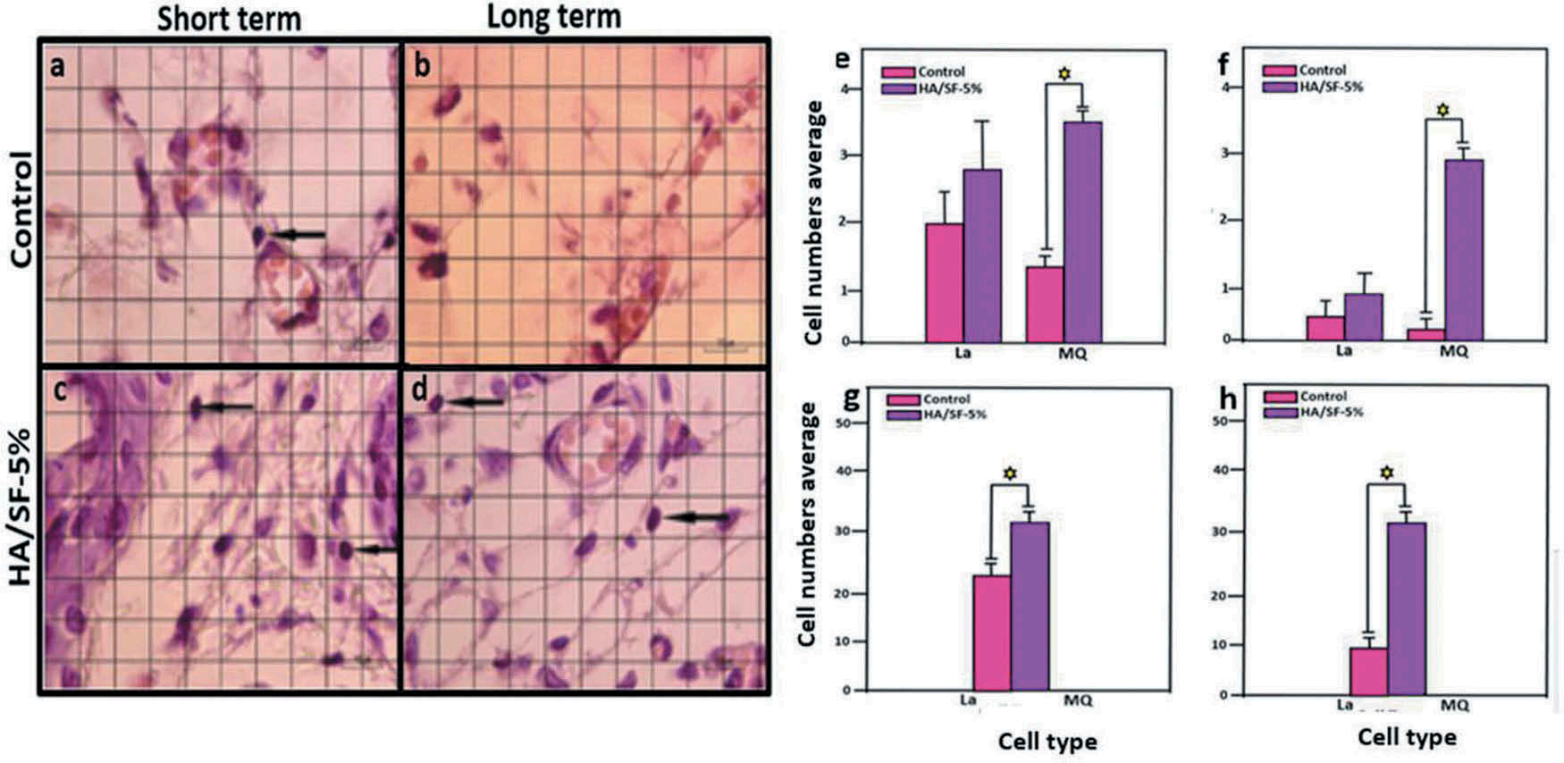
(a–d) *In vivo* biocompatibility of HA/SF-5% (black arrows denote lymphocytes, eosin and haematoxylin staining). (e–h) Increment of lymphocytes at the implantation site between control and experimental groups [187]

Many studies present HA as a promising biomaterial for bone tissue engineering. Despite of the fact that HA is a slowly biodegradable, this biomaterial displays high biocompatibility. Moreover, the biocompatibility of SF has also been proven by many studies [8,40,126,142,145]. More recently *Kweon* et al. have synthesized a nanoporous SF/HA composite and tested its biocompatibility *in vivo* [186].In another post-implantation study, in rat models the immune response against HA/SF‐5% (% of fibroin in aquatic solutions) is evaluated after long term (4 weeks) and short term (1 week). The number of inflammatory cells is analysed and counted including lymphocytes, macrophage, and cellularity at the tissue-scaffold interface area. Regarding the degradation process of the scaffold, the immunological attack and number of lymphocytes offer a valuable biocompatibility *in vivo*. As shown in Figure 22, the number of lymphocytic cells did not increase significantly as compared to control group (*P* > 0.05) in both short-term and long-term tests [187].

In contrast to the control experiment, a remarkable increase in number of macrophages has been observed. Macrophages may help in degradation process. Moreover, the prominent increase in the cellularity in the implantation area indicates the progress in tissue regeneration and degradation of scaffold [187].

**Figure 23.**
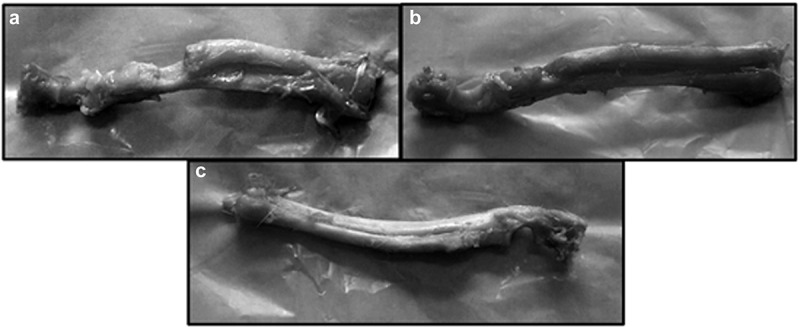
Photographs of rabbit bones on 12th week showing (a) no new bone formation without SF/HA-3, (b) new bone formation at bone defect site with SF/HA-3, and (c) new cortical bone formation and remodelling site with SF/HA-3 consisting BMSCs [188]

Wang et al. studied segmental bone defect by varying SF-to-HA molar ratios of the SF/HA composite. Four types of SF/HA composites with different SF weight loadings, different porosity, pore sizes, and additives were embedded subcutaneously into Sprague-Dawley rats to analyse biodegradation. It was observed that among four groups of SF/HA composite, SF/HA-3 is more suitable for bone substitute on the basis of strength and resorption, since SF/HA-1,SF/HA-2 were not degradable and SF/HA-4 have poor mean compressive strength of 1.59 MPa [188]. So, in order to evaluate its bone regeneration capacity, SF/HA-3 was selected as scaffold and co-cultured with rabbit bone-marrow stromal cells (BMSCs). It is found that this scaffold alone has no bone-inductive effect and limits bone repair efficacy. However, better results are observed with SF/HA-3 consisting BMSCs. (Figure 23(a–c)).

**Figure 24.**
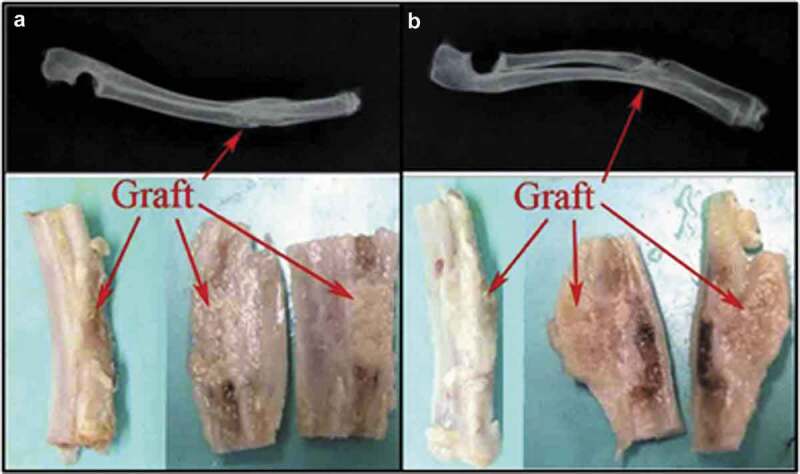
Radiographic illustration of gross anatomy of transplanted bone: structure of the defective radius at 12 weeks after operation in the experimental group (a) and the control group (b). Arrow indicates the transplantation site [189]

Similarly, Jin et al. investigate the potential of SF/HA scaffold as a delivery vehicle in the rabbit radius defect model for human placenta-derived mesenchymal stem cells (PMSCs). They subject transplantation of SF/HA alone (control group) to 16 New Zealand healthy rabbits and also SF/HA plus PMSCs (experimental group). Through histological and radiographic analyses, they show that fracture healing in the experimental group is significantly improved over the control group (Figure 24). This strongly suggests that the transplantation of human PMSCs, which are grown in an SF/HA scaffold into injured radius segmental bone in rabbits, can markedly enhance tissue repair [189].

## 5. Conclusions

This review suggests that due to increasing number of accidents and bone injuries, a large amount of strong and clinically safe biomaterials is required. HA/polymer scaffolds have been studied for their biocompatibility and osteoconductivity. Natural waste, such as eggshell and mint, etc., can be used to prepare HA. SF/HA scaffolds are one of the best options that can reduce risks during bone implantation owing to their exceptional bioactivity, proliferation activity and osteointegrativity. SF/HA scaffold is a cost effective precursor, because SF and HA are natural and readily available materials. SEM, XRD and FTIR absorption are important techniques for studying the resemblance of biomaterials with natural HA. ALP and proliferation activity studies can be very helpful in analysing SF/HA homogeneity and satisfying mechanical characteristics and biocompatibility. SF/HA-based composites are promising biomaterials, and researchers should continue exploring their diversity aiming to develop novel biomaterials.
